# Silymarin in combination with hair follicle transplantation as a potential treatment for refractory vitiligo: A double‐blind randomized controlled trial

**DOI:** 10.1111/jocd.16525

**Published:** 2024-08-20

**Authors:** Amir Feily, Masoome Hosseinpour, Leila Samipour, Seyedeh Yasamin Parvar, Maryam Hadibarhaghtalab, MReza Goodarzian

**Affiliations:** ^1^ Skin and Stem Cell Research Center Tehran University of Medical Sciences Tehran Iran; ^2^ Department of Otorhinolaryngology Mashhad University of Medical Sciences Mashhad Iran; ^3^ Student Research Committee Shiraz University of Medical Sciences Shiraz Iran; ^4^ Molecular Dermatology Research Center, Dermatology Department Shiraz University of Medical Sciences Shiraz Iran

**Keywords:** clinical trial, milk thistle, silymarin, *Silybum marianum*, vitiligo

## Abstract

**Background:**

There is still no certain effective treatment for vitiligo as a common chronic skin disorder characterized by depigmented patches and loss of skin melanocytes.

**Objectives:**

This study evaluates the efficacy of oral silymarin combined with hair follicle transplantation compared to follicle transplantation alone in the treatment of refractory vitiligo.

**Materials and Methods:**

Twenty refractory vitiligo patients were enrolled in this randomized controlled clinical trial, following up for 3 months. One group underwent hair transplantation plus oral silymarin, while the other group underwent follicle transplantation alone. We assessed the progress with Vitiligo Extent Tensity Index (VETI) in both groups and the peri‐follicular pigmentation diameter was estimated monthly. The Friedman test for comparing two groups at the end and the Mann–Whitney test for comparing two groups during each month was used.

**Results:**

The mean age was 30.22 (18–59) years, with the male to female ratio of 1:1. The decrease in the VETI and increase in the perifollicular pigmentation was statistically significant between silymarin and another group in monthly follow‐up (*p*‐value: 0.019, 0.019, and 0.035, respectively). Finally, the re‐pigmentation was notable in silymarin group (*p*‐value <0.001 vs. 0.029, respectively). In addition, both genders had a significant increase in peri‐follicular re‐pigmentation in the last follow‐up (*p*‐value: 0.012 and 0.044, respectively); although the improvement was not statistically significant between genders in each month.

**Conclusion:**

According to our study, silymarin in combination with hair transplantation could be a potential medical treatment for vitiligo; however, further trials are needed to establish the efficacy of combination therapies.

## 1 INTRODUCTION

Vitiligo is a common skin depigmentation disease that manifests as well‐defined white macules and patches. It is characterized by the gradual loss of melanocytes in the epidermis and hair follicles.^1^, ^2^ Vitiligo affects approximately 0.1%–2% of the general population. Additionally, it affects females and youth.^3^

Vitiligo has psycho‐dermatologic aspects and it would affect self‐confidence and beauty at the same time. Therefore, a higher level of depression and anxiety is observed among these participants, which in turn, compromises the quality of life.^4^

Different surgical and medical options like punch grafting and topical trioxsalen are available for the treatment of vitiligo.^5^ These options stabilize the active illness, restore the depigmentation, and return the psycho‐social problems in order to achieve acceptable cosmetic and confidence outcomes.^4^, ^6^, ^7^ Recent time‐consuming treatments and the diversity of outcomes among individuals have made them unsatisfactory. As a result, new therapeutic approaches are required to tackle vitiligo.^1^, ^8^ Hair follicle transplantation is a safe and effective treatment for localized and segmental vitiligo, particularly in hairy areas. The hair follicle contains undifferentiated stem cells, which are reliable sources of melanocytes for re‐pigmentation. After a few weeks, melanocytes form in the depigmented epidermis around the transplanted follicle, and the skin appears re‐pigmented.^7^, ^9^, ^10^, ^11^, ^12^ Due to short recovery, low morbidity, and acceptable color match, hair follicle transplantation was considered the procedure of choice in focal vitiligo with scarring or hair loss in various trials.^9^, ^12^

On the other hand, silymarin, a flavonoid with anti‐oxidant properties extracted from milk thistle (*Silybum marianum*), is a potent immune‐modulator and anti‐oxidant medical choice.^13^, ^14^ The effect of silymarin on different dermatological conditions such as melasma^15^ and acne‐vulgaris^16^ has been conducted but there are few trials performed on the therapeutic effect of silymarin on vitiligo.^17^ The current study aims to assess the efficacy of silymarin plus hair follicle transplantation in comparison with hair follicle transplantation alone on participants diagnosed with refractory vitiligo.

## 2 METHODS AND MATERIALS

### 2.1 Design of the study

This retrospective double‐blind randomized controlled trial was performed at Jahrom Dermatology Outpatient Clinic, one of the academic referral centers for dermatology in the South of Iran. The study was performed according to the Helsinki Declaration and approved by the Ethics Committee of the University. Written informed consent was obtained from all participants before the study and the trial was registered at the Iranian Registry of Clinical Trials (IRCT2017052925407N3).

### 2.2 Inclusion and exclusion criteria

During 10 months of examination, 20 participants diagnosed with non‐segmental refractory (did not respond to the previous common treatments) and stable (without new lesions) vitiligo lesions were enrolled in the study. The procedures and probable adverse effects were explained to each participant before the study and were evaluated after each visit. At baseline, demographic characteristics were obtained from participants. The inclusion criteria consist of all refractory vitiligo lesions existing for at least 6 months in participants aged over 18 years, involving 15%–50% of the Total Body Surface Area (TBSA), and Fitzpatrick skin type III and VI confirmed by physical examination and Wood's lamp.

The exclusion criteria were vitiligo lesions presenting on joints and mucus membrane, participants with leukotrichia, pregnant and lactating women, individuals with impaired liver function and renal failure, participants on topical or oral medication for vitiligo, existing comorbidities including active systemic inflammation, active infection on the scalp, active trichotillomania, and bleeding disorders, those with hypersensitivity reactions to silymarin, and lack of informed consent.

### 2.3 Randomization and blindness

The Random Allocation Software Inc. (version 1.0, May 2004) was used to create a randomization table with a block size of four. Based on the randomization table, the participants were assigned to two groups with 1:1 allocation rate. Moreover, a biostatistician who analyzed the data was also blinded to the two groups.

### 2.4 Intervention and assessment

In the beginning, the disease severity was determined based on the Vitiligo Extent Tensity Index (VETI).^15^ The participants were randomly enrolled in two groups; silymarin plus hair follicle transplantation versus hair follicle transplantation alone. Ten participants in the first group received hair transplantation along with 70 mg of silymarin (Gol Daru Co., Livergol 70 mg oral tab, Iran) twice a day for 3 months; while 10 participants in the second group received hair transplantation alone. Then, all the participants were referred for phototherapy with Narrow‐Band Ultra‐Violet B (NBUVB) (Dermfix Ltd) three times a week. According to the previous studies regarding vitiligo interventions and treatments,^18^, ^19^ the treatment course was set 3 months and participants were visited monthly to measure peri‐follicular re‐pigmentation diameter with calipers. The participants were regularly checked for any grafting side effects (visible or hypertrophic scarring, keloid formation, effluvium of the grafted area, and cobble‐stoning around the hair grafts).

### 2.5 Statistical analysis

Data were analyzed using IBM Statistical Package for the Social Sciences (version 26.0 for Windows; SPSS Inc., Chicago, IL, USA). The Shapiro–Wilk test was used to evaluate the normality, the Mann–Whitney test was used to compare the means between two independent groups (silymarin plus hair follicle transplantation vs. hair follicle transplantation and male vs. female) at the end of each month, and the Friedman test was used to compare means between two groups at the end of the follow‐up. Descriptive data were expressed as mean ± standard deviation and percentage. *p*‐value less than 0.05 was considered significant.

## 3 RESULTS

**FIGURE 1:**
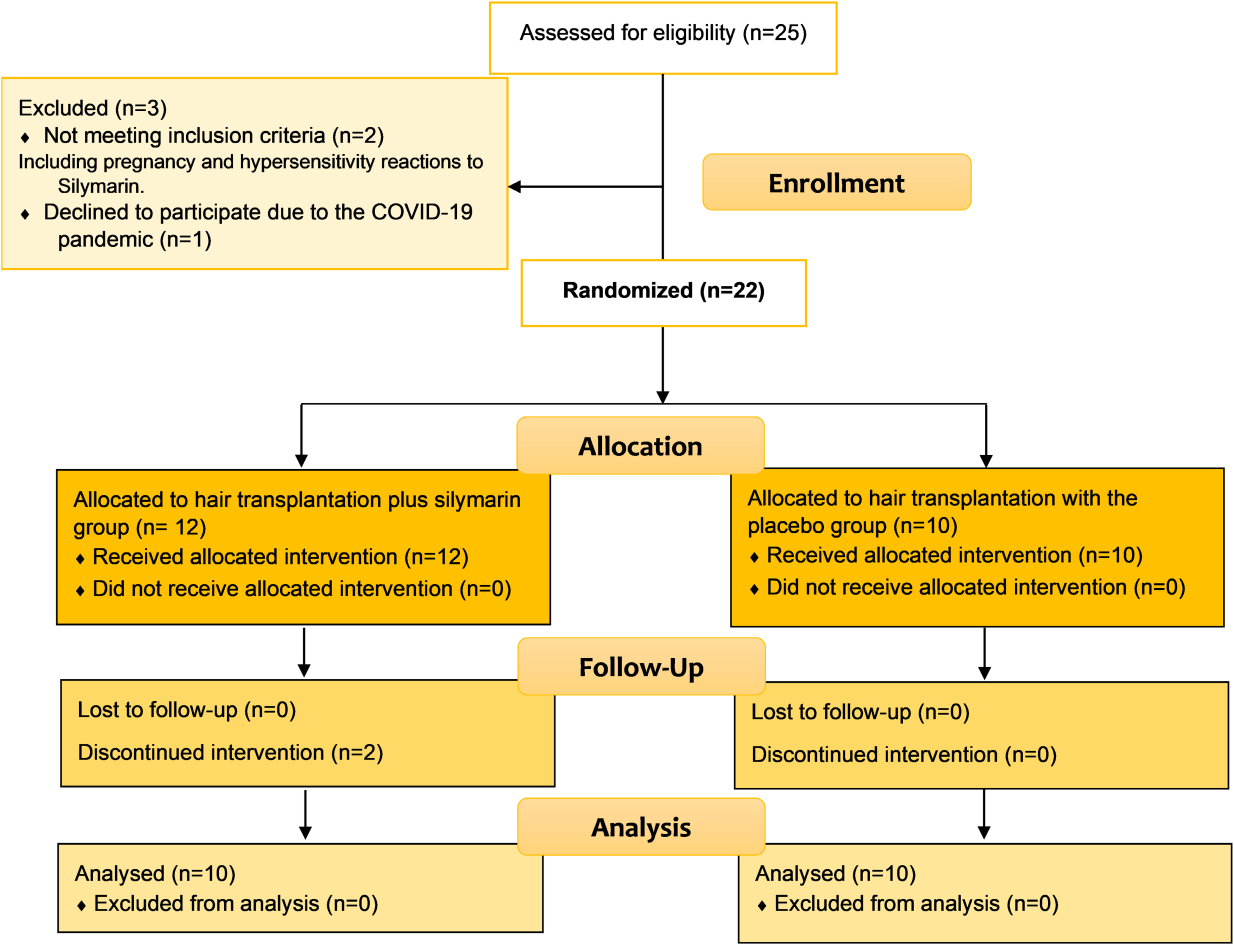
Consort flow diagram of the trial showing the selection, randomization, allocation, follow‐up, and analysis of the participants.

A total number of 25 participants were assessed for eligibility. Two participants were excluded due to the pregnancy and hypersensitivity reactions to silymarin and three of them discontinued intervention or declined to participate (Figure 1). Among the 20 participants in the trial, male to female ratio was 1:1 and the mean age was 30.22 years old, ranging from 18 to 59 years. There was no statistically significant difference in perifollicular pigmentation diameter between the two groups at the beginning of the study.

As shown in Table 1, there was an increase in the peri‐follicular re‐pigmentation in both groups, which was statistically significant (*p*‐value <0.001 and 0.029, respectively). The increase was more notable in the silymarin plus hair follicle transplantation compared with the hair follicle transplantation alone group at the end of the 3 months' trial. There was also a significant difference in the mean pigmentation in first, second, and third month of follow‐up between both groups (*p*‐value: 0.019, 0.019, and 0.035, respectively).

**TABLE 1:** Comparison of the mean peri‐follicular re‐pigmentation between silymarin plus hair follicle transplantation and hair follicle transplantation groups during 3 months follow‐up; mean ± SD.

Follow‐up period	Group		*p*‐value* (between groups)
	Silymarin plus hair follicle transplantation (*N* = 10) (Mean ± SD^a^)	Hair follicle transplantation (*N* = 10) (Mean ± SD)	
Baseline	0.00 ± 0.0	0.0 ± 0.0	
1st month	0.46 ± 0.5	0.0 ± 0.15	0.019
2nd month	0.60 ± 0.83	0. 0 ± 0.33	0.019
3rd month	0.64 ± 1.66	0.0 ± 0.56	0.035
Trend *p*‐value**	*p* ≤ 0.001	0.029	

^a^ Standard deviation.

* According to the Mann–Whitney test.

** According to the Friedman test.

Table 2 compares peri‐follicular pigmentation in the silymarin plus hair follicle transplantation group during a three‐month follow‐up based on gender. Males had clinically higher mean re‐pigmentation during each visit compared to females, although it was not statistically significant. It is also worth mentioning that males and females in the silymarin plus hair follicle transplantation groups showed significant improvement at the end of the three‐month follow‐up (*p*‐values: 0.012 and 0.044, respectively). It is noteworthy that the grafting complication was seen only in one patient which was insignificant.

**TABLE 2:** Comparison of peri‐follicular re‐pigmentation in the silymarin plus hair follicle transplantation group during three‐months follow‐up based on gender.

Follow‐up period	Male (*N* = 10) (Mean ± SD^a^)	Female (*N* = 10) (Mean ± SD)	*p*‐value* (between genders)
Baseline	0.00 ± 0.0	0.0 ± 0.0	
1st month	0.50 ± 0.08	0.42 ± 0.50	0.421
2nd month	0.83 ± 0.23	0.50 ± 0.60	0.222
3rd month	1 ± 1.02	0.50 ± 0.64	0.310
Trend *p*‐value**	0.012	0.044	

^a^ Standard deviation.

* According to the Mann–Whitney test.

** According to the Friedman test.

## 4 DISCUSSION

The current randomized controlled clinical trial compared the efficacy of hair follicle transplantation plus silymarin versus hair follicle transplantation alone, as a current treatment option, on participants diagnosed with refractory vitiligo with a mean age of 30 years. Our findings showed that there was an improvement in depigmented areas based on the VETI score in both groups after three‐month of treatment period without serious grafting complications.

Vitiligo is a severe chronic and acquired skin pigmentation disorder that significantly impacts the quality of life. The psychological burden of vitiligo is substantial, often leading to decreased self‐esteem, social stigma, and a higher prevalence of depression and anxiety. Effective treatments that provide variable degrees of re‐pigmentation^8^ can therefore have profound benefits on both physical appearance and psychological health.^4^ The treatment has remained a challenge, owing to the complexities of its pathophysiology.^8^, ^20^

Silymarin, a powerful antioxidant and immune‐modulatory agent, increases intracellular glutathione levels, inhibits the generation and secretion of Th1 cytokines, nitric oxide, and nuclear factor kappa B, and suppresses tumor necrosis factor and T‐cell receptor synthesis.^13^, ^14^, ^21^, ^22^ silymarin also inhibits Cyclic Adenosine Mono Phosphate (cAMP) phosphodiesterase; Thus, raises intracellular cAMP levels. The cAMP surge, directly or indirectly, and the inhibition of phosphatidylinositol‐3‐kinase (PI3K), increases melanogenesis. Increased intracellular cAMP promotes protein kinase‐A enzyme, which activates the Melanocyte‐inducing Transcription Factor (MITF); hence, tyrosine gene expression enhances and contributes to melanocytic stem cell proliferation. Meanwhile, cAMP activates Glycogen Synthase Kinase 3 Beta, which promotes tyrosine gene expression through MITF phosphorylation. These actions contribute to its potential therapeutic effects in vitiligo by reducing oxidative stress and modulating immune responses.^14^, ^23^, ^24^

In 2006, silymarin was licensed as a skin pigmentation promotor.^25^ In 2009, Sehgal investigated the use of tacrolimus ointment combined with trioxsalen and silymarin suspension in treating progressive vitiligo in eight children. Six months follow‐up showed the effective treatment of vitiligo lesions.^6^ Similarly, Feily et al. in 2011 hypothesized the therapeutic potential of silymarin in vitiligo due to its biochemical properties, and Mehraban et al. in 2019 reviewed various dermatological applications of silymarin, including its potential benefits in treating conditions like melasma, acne, and vitiligo.^14^, ^17^ Jowkar et al. conducted a double‐blind Randomized controlled Clinical Trial (RCT) to evaluate the efficacy of oral silymarin in combination with phototherapy in 34 patients with a mean age of 34 years and found that silymarin plus phototherapy did not significantly improve the vitiligo lesions.^26^ The current study's findings suggested that hair transplantation alone could be an effective treatment of vitiligo; however, when combined with *S. marianum*, the treatment is more efficient.

Additionally, other studies have highlighted the broader dermatological benefits of silymarin. Kim et al. assessed the efficacy and safety of a silymarin‐loaded antioxidant serum in treating mild‐to‐moderate acne vulgaris, reporting significant improvements in acne severity, melanin pigmentation, and sebum secretion, with no adverse events.^16^ Toklu et al. demonstrated the protective effects of silymarin against oxidative skin injury induced by burns, highlighting its strong antioxidant properties.^13^

We believe that the present study's findings would expand current knowledge about the importance of vitiligo management as it affects both an individual's appearance and self‐estimation. Our study had several limitations. First, the small sample size may limit the generalizability of our findings. Future research with larger populations, dose assessments, and topical formulations of *S. marianum* are needed to confirm and expand upon our results. Furthermore, due to the lack of the patient's consent regarding sharing their photos, we were unable to include photos of their vitiligo lesions during the treatment course.

## 5 CONCLUSION

In summary, the current trial demonstrates the efficacy of adding oral silymarin to hair follicle transplantation in vitiligo‐diagnosed participants. Therefore, silymarin can be considered as a new therapeutic option for vitiligo; nevertheless, further trials are needed to establish the efficacy of combination therapies for vitiligo.

## AUTHOR CONTRIBUTIONS

A.F. contributed to study conception and design, M.H. to data acquisition, L.S. to data analysis and interpretation, S.Y.P. to manuscript drafting and critical revisions, M.H.B. to study conception and design, and M.R.G. to data acquisition and manuscript drafting. All authors have approved the final version of this manuscript.

## FUNDING INFORMATION

No funding.

## CONFLICT OF INTEREST STATEMENT

The authors declare no conflicts of interest.

## ETHICS STATEMENT

The Medical Ethics Committee of the university and the Iranian Registry of Clinical Trials (IRCT2017052925407N3) approved the present study according to the declaration of Helsinki. We confirm that all figures and tables are original and created by authors. We would also like to mention that we have read the plagiarism policy and submitted the article with complete responsibility. The informed consent was acquired during the first visit and the purpose of this research was thoroughly explained to the participants. They were assured that their information would be kept confidential by the researcher. Ethics Committee of the university approval number: IR.JUMS.REC.1394.183.

## TRANSPARENCY STATEMENT

The corresponding author Dr. Maryam Hadibarhaghtalab affirms that this manuscript is an honest, accurate, and transparent account of the study, that no important aspects of the study have been omitted, and that any discrepancies from the study as planned have been explained.

## Data Availability

The data that support the findings of this study are available from the corresponding author upon reasonable request.
